# Supplementary material for: Reduced Cross-Sectional Muscle Growth Six Months after Botulinum Toxin Type-A Injection in Children with Spastic Cerebral Palsy
Source: Toxins (Basel). 2022 Feb 14;14(2):139. doi: 10.3390/toxins14020139 (PMC8876834; doi:10.3390/toxins14020139)
Supplement: Supplementary file 1 [file toxins-14-00139-s001.zip › toxins-1587387-supplementary.pdf]

Supplementary Materials: Reduced Cross-Sectional Muscle Growth Six Months after Botulinum Toxin Type-A Injection in Children with Spastic Cerebral Palsy

Nathalie De Beukelaer, Guido Weide, Ester Huyghe, Ines Vandekerckhove, Britta Hanssen, Nicky Peeters, Julie Uytterhoeven, Jorieke Deschrevel, Karen Maes, Marlies Corvelyn, Domiziana Costamagna, Ghislaine Gayan-Ramirez, Anja Van Campenhout and Kaat Desloovere

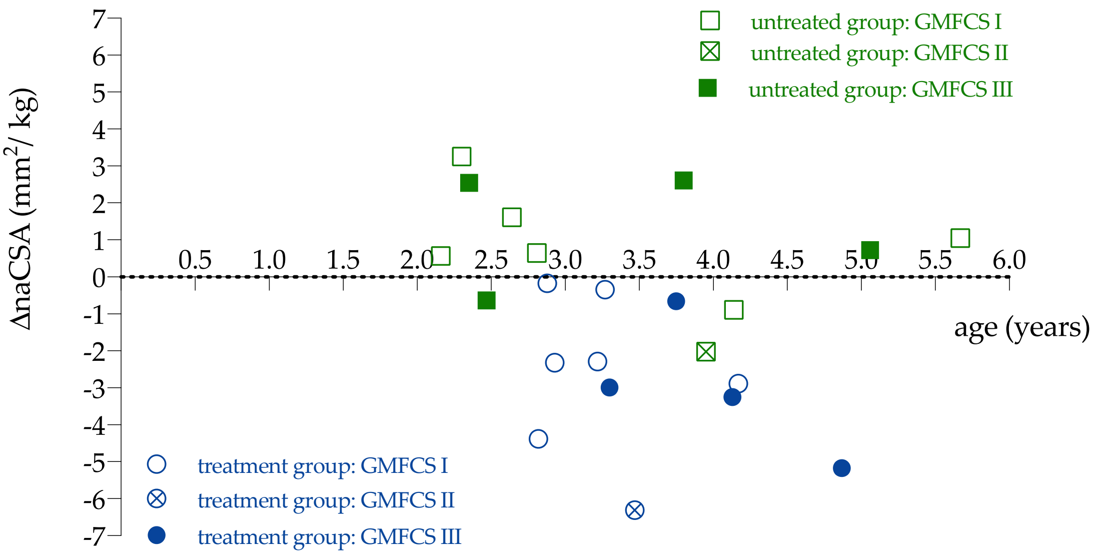

Figure S1. Individual data of the change in normalized anatomical cross-sectional area per age in the *botulinum neurotoxin type-A naive treatment* and *untreated group*, visualized per GMFCS level. *naCSA, normalized anatomical cross-sectional area; mm, millimeter; kg, kilogram; GMFCS, gross motor function classification scale*

Table S1. Usual care, functional mobility and clinical examination of the ankle joint for the *BoNT-A naive treatment*, *BoNT-A naive untreated* and *BoNT-A not-naive treatment* group at the time of the baseline assessment.

	**BoNT-A naive treatment group**	**BoNT-A naive untreated group**	**BoNT-A not-naive treatment group**
Participants (n)	11	11	15
Use of orthoses during the day	Frequently used, *n* = 10	Frequently used, *n* = 9	Frequently used, *n* = 13
	Not frequently used, *n* = 1	Not frequently used, *n* = 2	Not frequently used, *n* = 1
	Not used, *n* = 0	Not used, *n* = 0	Not used, *n* = 1
Use of orthoses during the night	Frequently used, *n* = 0	Frequently used, *n* = 0	Frequently used, *n* = 2
	Not frequently used, *n* = 4	Not frequently used, *n* = 3	Not frequently used, *n* = 4
	Not used, *n* = 7	Not used, *n* = 8	Not used, *n* = 9
Physiotherapy, min per week	120 (60 to 230)	90 (60 to 270)	120 (60 tot 240)
Oral medication	Tone reduction, *n* = 2	Tone reduction, *n* = 1	Tone reduction, *n* = 4
	Anti-epileptics, *n* = 1	Anti-epileptics, *n* = 2	Anti-epileptics, *n* = 0
	Others, *n* = 1	Others, *n* = 4	Others, *n* = 0
	Not used, *n* = 8	Not used, *n* = 3	Not used, *n* = 11
Functional mobility scale	5m: 5 (1-6); 50m: 5 (1-6); 500m: 5 (1-6)	5m: 5 (1-6); 50m: 5 (1-6); 500m: 5 (1-6)	5m: 6 (5-6); 50m: 5 (2-6); 500m: 5 (1-6)
Ankle joint mobility	Normal, *n* = 8	Normal, *n* = 6	Normal, *n* = 5
	Mild limitation, *n* = 3	Mild limitation, *n* = 5	Mild limitation, *n* = 7
	Contracture, *n* = 0	Contracture, *n* = 0	Contracture, *n* = 3
Plantar flexor muscle spasticity	Normal to mild, *n* = 1	Normal to mild, *n* = 4	Normal to mild, *n* = 1
	Moderate, *n* = 6	Moderate, *n* = 6	Moderate, *n* = 11
	Severe, *n* = 4	Severe, *n* = 1	Severe, *n* = 3

Frequently used indicated ≥ 50% of the day or night whereas not frequently used indicated <50% of the day or night. The amount of physiotherapy and the sub-scores of functional mobility scale are ~~is~~ shown as median (minimum to maximum values). Mobility was classified as no contracture (≥10 degrees of dorsiflexion), mild restriction in ROM (10 to 0 degrees of dorsiflexion) and contracture (< 0 degrees of dorsiflexion). Spasticity was classified as normal to mild increased tone (MAS of 0 or 1), moderate spasticity (MAS of 1+ or 2) and severe spasticity (MAS of 3). The most involved leg is reported. *BoNT-A, botulinum neurotoxin type A; n, number.*

Table S2. BoNT-A treatment doses (units/body mass) in the *BoNT-A naive treatment* and *BoNT-A not-naive treatment* group

The doses expressed as units (U) per kg body mass are shown as median (minimum to maximum values). *BoNT-A, botulinum neurotoxin type A; n, number; NA, not applicable.*

	**BoNT-A naive treatment group**		**BoNT-A not-naive treatment group**	
	**Participants (n)**	**Dose (U/kg)**	**Participants (n)**	**Dose (U/kg)**
Total	11	8.0 (4.4–10.8)	15	7.9 (4.4–10.9)
Medial gastrocnemius	11	2.7 (0.9–4.1)	15	2.7 (1.7–4.3)
Psoas	6	1.4 (0.9–1.6)	2	0.8 (0.8–0.9)
Rectus femoris	0	NA	3	1.5 (1.2–1.8)
Semitendinosus	10	2.9 (1.5–5.9)	13	1.8 (1.1–3.8)
Semimembranosus	5	1.3 (0.7–1.8)	13	1.4 (0.9–2.3)
Gracilis	6	1.9 (1.5–2.7)	11	1.5 (1.0–2.6)
Adductors	2	0.7 (0.7–0.8)	1	1.1
Soleus	1	2.2	7	1.7 (1.0–2.3)

Table S3. Comparison of median group values for anthropometric measures and normalized medial gastrocnemius muscle outcomes between the *BoNT-A treatment groups*, *BoNT-A naive untreated* and *TD group* at the time of the baseline assessment.

Baseline	BoNT-A naive treatment (a)	BoNT-A naive untreated (b)	BoNT-A not-naive treatment (c)	TD (d)	*p*	a-d, *p*	b-d, *p*	c-d, *p*
Participants (n)	11	11	15	49				
Age (y)	3.3 (1.2)	2.8 (1.8)	6.4 (2.5)	5.5 (3.7)	<0.001	0.008	0.004	0.453
Body mass (kg)	13.6 (1.3)	14.5 (4.9)	20.0 (7.5)	20.0 (6.9)	<0.001	0.000	0.004	1.000
Body length (cm)	91.0 (9.20)	97.0 (18.4)	114.0 (26.0)	112.0 (21.6)	<0.001	0.000	0.001	1.000
Leg length (cm)	44.2 (5.0)	46.0 (9.5)	55.4 (15.5)	55.0 (14.3)	<0.001	0.001	0.003	1.000
nMV (mL/(kg·m))	1.47 (0.43)	1.52 (0.45)	1.39 (0.62)	1.78 (0.40)	<0.001	0.003	0.060	0.003
nML (%)	23.6 (4.05)	23.3 (5.82)	23.4 (3.46)	26.70 (3.85)	<0.001	0.031		0.002
nMTUL (%)	45.8 (1.92)	46.7 (5.55)	45.5 (1.49)	46.88 (2.45)	0.001	0.204		0.006
aCSA$_{norm}$ (mm^2/kg)	16.7 (6.40)	17.5 (4.15)	17.8 (5.56)	21.79 (3.82)	<0.001	0.001	0.001	0.000
EI (bit)	153.2 (12.1)	158.5 (31.5)	175.3 (22.4)	152.3 (19.9)	0.004	1.000	1.000	0.002
Growth rate (mL/mo)	0.47 (0.25)	0.51 (0.09)	0.42 (0.21)	0.58 (0.18)	<0.001	0.003	0.344	0.000

Data are presented as median (interquartile range). Muscle growth rate was calculated as ratio of muscle volume (milliliter) per age (months). Kruskall-Walis test with pairwise post-hoc analysis and significance level of 0.01 after Bonferroni correction was used. Significant results were indicated with grey color. *BoNT-A, botulinum neurotoxin type A; TD, typically developing; n, number; y, years; kg, kilograms; cm, centimeter; nMV, muscle volume normalized to the product of body mass and body length; mL, milliliter; m, meter; nML, muscle belly length normalized to leg length; nMTUL, muscle-tendon unit complex length normalized to leg length; aCSA$_{norm}$, anatomical cross-sectional area normalized to body mass; EI, echo-intensity; mo, month.*
